# Menopause-Related Symptoms and Influencing Factors in Mosuo, Yi, and Han Middle-Aged Women in China

**DOI:** 10.3389/fpsyg.2022.763596

**Published:** 2022-06-10

**Authors:** Jinyi Wang, Yezhe Lin, Limin Gao, Xingjun Li, Chunhua He, Maosheng Ran, Xudong Zhao

**Affiliations:** ^1^School of Humanities, Tongji University, Shanghai, China; ^2^Clinical Research Center for Mental Disorders, Shanghai Pudong New Area Mental Health Center, School of Medicine, Tongji University, Shanghai, China; ^3^Department of Psychosomatic Medicine, Shanghai East Hospital Affiliated to Tongji University, Shanghai, China; ^4^Tongji University School of Medicine, Shanghai, China; ^5^Department of Rheumatology and Immunology, First Affiliated Hospital of Kunming Medical University, Kunming, China; ^6^The Second People’s Hospital of Lijiang, Lijiang, China; ^7^Mental Health Center, West China Hospital, Sichuan University, Chengdu, China

**Keywords:** Mosuo women, Yi women, female status, family support, cross-cultural comparison

## Abstract

Although previous studies showed that women’s menopause-related symptoms varied in different ethnic groups and countries, and were affected by specific social and cultural factors, few studies have been conducted to explore menopause-related symptoms and its influencing factors in middle-aged women among ethnic groups in China. This study aimed to explore the characteristics of menopause-related symptoms and its influencing factors among Mosuo, Yi, and Han women in Yongning area of Yunnan province, China. A cross-cultural design by snowball sampling method was used to recruit 208 women aged 40–60 from Yongning Township, Ninglang County, Yunnan province, China. The 11-item Menopause Rating Scale (MRS) was used to assess menopause-related symptoms. Compared with Yi and Han women, Mosuo women were accorded the highest family status. Multiple linear regression analyses showed that ethnicity, age, family support, and family decision-making patterns were associated with the severity of menopause-related symptoms. Yi and Han women had more severe menopause-related symptoms than Mosuo women. Among the three groups, women living in “female-dominated” and “co-deliberated” households had significantly lower scores of menopause-related symptoms than those in “male-dominated” households. This study indicates that menopause-related symptoms vary among middle-aged women in different ethnic groups. A higher level of female status in the family and family support may be protective factors of menopause-related symptoms in middle-aged women.

## Introduction

Culture, which accounts for everything that is not biologically determined (Flint and Samil, 1990), affects and shapes people’s mentality and behavior implicitly. The manifestations of many mental disorders or symptoms (e.g., somatization, postpartum depression, and menopause-related symptoms) are also modified by cultural factors (Melby et al., 2005; Halbreich and Karkun, 2006; Zhou et al., 2016). Middle-aged women go through menopause (also known as climacteric), a normal transition from the reproductive to the non-reproductive phase (WHO Scientific Group, 1996). During this period, women often experience a series of physical and psychological symptoms, previously named menopause syndrome, including menstrual disturbances, hot flashes, sweating, palpitations, insomnia, depression, agitation, and irritability (Chinese Medical Doctor Association, 2021). Prior studies showed that women’s menopausal symptoms were related to the hypothalamic-pituitary-ovarian axis (Zhang and Yan, 2021), endocrine dysfunction, changes in social roles, and increased risk of physical and emotional problems (Greendale et al., 1999). Given that the etiology is multifactorial and multidimensional, menopausal syndrome is also considered as a psychosomatic disorder (Facchinetti et al., 1992).

A large-scale transcultural study of women aged 40–60 showed that participants from different countries and ethnic groups experienced different psychosomatic symptoms of menopause (Shea, 2006). Some anthropologists suggest that the “menopausal symptomatology” may well be culturally defined and engendered (Flint, 1975). Specific socio-cultural factors such as the meaning of menopause, family relationships, family support, developmental environment during childhood, and religion play a role in the development of menopause-related symptoms (Parkerson et al., 1989; Obermeyer, 2000; Begum et al., 2016; Zhao et al., 2019). Studies conducted in Tehran (Movahed et al., 2015), Northern Iran (Abasi et al., 2020) and the United States (Wu and Sheng, 2019) reported that individuals having a higher level of social support experienced less physical and mental disturbances. Although many cross-cultural studies show that age and menopause-related symptoms might be different in various ethnic groups, few studies have been conducted to explore menopause-related symptoms and its influencing factors in different ethnic groups in China (Obermeyer, 2000; Avis et al., 2001; Hvas et al., 2003; El Khoudary et al., 2019).

China comprises 56 ethnic groups, which have formed distinct ethnic cultures in the historical development of the country. Yunnan, in the southwest of China, is the most ethnically diverse region among the whole nation. It is home to two unique ethnic groups, Mosuo and Yi, representing the matrilineal and patrilineal societies, respectively (Gong and Yang, 2012). Mosuo people are self-proclaimed as “Na” or “Nari” people who live in the junction of Sichuan and Yunnan, such as Ninglang Yi Autonomous County of Yunnan Province, and Yanyuan County, Muli Tibetan Autonomous County, Panzhihua City, and Yanbian County of Sichuan Province (HasErdene, 2018). For Chinese nationality identification, Mosuo were spilt into two groups based on geography. The clan of Mosuo in Yunnan Province was classified as the Naxi and the other in Sichuan Province was called Menggu (also called “Mongolian”). Mosuo, regarded as the “living fossils” of matriarchy (Yan, 1984; Sum et al., 2021), has diverged from original Naxi culture (Harrell, 2001). Therefore, the term “Mosuo” refers to this particular group.

The Mosuo group’s ancestors were the ancient Qiang people of the Huang generation, who moved south into Liangshan Prefecture during the Warring States period and later settled down. Mosuo people are famous for their extended matrilineal families and traditional Mosuo marriage is known as “Tisese” (translates as “walking marriages” or “visiting marriages”) (Guo, 2017), where the man and the woman stay in their mothers’ households when they are in a relationship. Men would visit the woman’s household at night. They were not bound by any economic obligations to each other (Cai, 2001). Historically, children were raised collectively by the woman’s family without the father. Nowadays, in Mosuo culture, some men have begun to live in the woman’s house, but they still belong to their mother’s household. Thus, this modern Mosuo marriage is still regarded as “Tisese.” Mosuo people give importance to their matrilineal lineage system, in which maternal kinship is crucial in determining family structure. The “grandmother” is the most respected person in the family and as the core of blood relations in a Mosuo family. She is also in charge of family daily life, such as family property, housework, and reception of guests. About 81% of Mosuo families in Yongning still belonged to matrilineal extended families in 2019 (Yang, 2020). The overall value is “respecting mother and the elderly” (Zhao, 2011), which indicates that Mosuo women’s status increases with age in such a culture.

Yi people are also descendants of the ancient Qiang; however, their ancestors transitioned to a patrilineal society more than 2,000 years ago. The Yi people were traditionally slash-and-burn farmers. Their society is structured by a patrilineal exogamous lineage called “Jiazhi” which is the family union of Yi with the paternal blood as the bond, and the father and son’s joint genealogy as the family chain. Yi women are not included in the pedigree, nor have the right to inherit any property (Liu, 2007). The culture of “Jiazhi,” that once played the role of political authority, still plays an important role among the Yi people. The nature and function of the contemporary Yi family is different from that of the past, given the change in history. Moreover, although the current status and situation of Yi women have improved slightly, they still lack authority in the family. In general, currently Yi women are in a subordinate position and are easily marginalized in the cultural pattern of family support (Hao, 2015). Compared to Mosuo and Yi, Han people have a relatively moderate gender culture. They have transitioned from a matrilineal to a patrilineal society since the Xia Dynasty (Feng, 2013). Since the establishment of the People’s Republic of China in 1949, the concept of patriarchal clan that used to be rooted in Han Chinese people has been dispersing to many central areas of China. Gender equality has been mentioned in the Chinese Constitution in 1954 (Zheng, 2020), and since then, gender equality has been gaining popularity among the population.

A study reported that Mosuo women might have milder menopause-related symptoms than Han women (Zhang et al., 2013). Although there are different gender concepts and family structures among Mosuo, Yi, and Han people, few studies have been conducted to explore the impact of these specific cultural factors (e.g., women’s family status, social support) on the manifestations of menopause-related symptoms among these three ethnic groups. Thus, this study aimed to explore the characteristics of menopause-related symptoms and its influencing factors among Mosuo, Yi, and Han women in Yongning area of Yunnan province, China. The hypotheses tested in this study are: (1) Mosuo, Yi, and Han middle-aged women have different levels of menopause-related symptoms; and (2) different family circumstances (e.g., women’s family status, social support) are associated with menopause-related symptoms.

## Materials and Methods

### Participants

From December 2019 to July 2020, a cross-section investigation was conducted in 21 villages in Yongning Township, Ninglang County, Yunnan Province. According to the population data of Yongning County police station, there were 2,038 women aged between 40 and 60, including 1,071 Mosuo, 316 Yi, 651 Han, and other ethnicities. Applying G Power software (Faul et al., 2007), we calculated the required sample size for Analysis of Variance (ANOVA) with an effect size of d = 0.25 [an error probability = 0.05, Power (1-βerr prob) = 0.9] and for a critical *F* = 3.0401584, df = 204, which resulted in a total sample size of 207 for three groups. The inclusion criteria for this study were: (1) Mosuo, Yi, and Han women aged 40–60; and (2) informed consent to participate in this research. The exclusion criteria for this study were: (1) Having non-natural menopause (such as caused by hysterectomy); (2) using hormones such as estrogen replacement or other drugs that might affect endocrinology (e.g., antidepressant and anxiety drugs, breast cancer drugs among others); (3) having a history of severe physical or mental illness; and (4) unable to complete the interview.

Limited by poor traffic in the field, snowball sampling was conducted in the 21 nearest villages in Yongning Township, and about ten women were to be recruited from each village. Among a total of 219 women (87 Mosuo, 72 Yi, and 60 Han) screened, 3 Mosuo, 7 Yi, and 1 Han women were excluded because of severe physical diseases or poor comprehension of the instructions. Eventually, 208 women (84 Mosuo, 65 Yi, and 59 Han) participated in this study. All participants who completed the study received a 30 RMB supermarket voucher or equivalent as compensation for buying daily necessities.

### Procedure

Given the different languages used by Mosuo, Yi, and Han people, the investigation was conducted through interpreters. Six women with bachelor’s degrees in medicine, nursing, psychology, or education were recruited as interpreters for the investigation (2 interpreters could understand and fluently speak the Mosuo language and Yunnan Chinese dialects, and 4 interpreters could understand and fluently speak the Yi language and Yunnan Chinese dialects). All the interpreters were trained to use the instruments before the investigation and met the training requirements. All the participants in this study were interviewed through field oral translation in order to maintain consistency in assessment methods. The investigative interview for each participant lasted for about an hour.

### Measures

**FIGURE 1:**
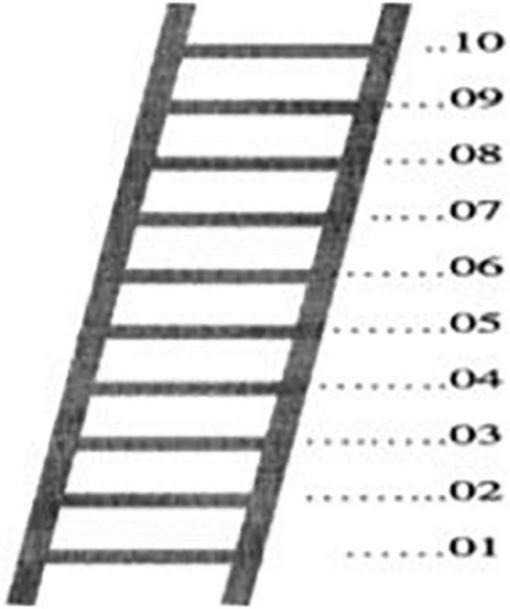
Cantril ladder.

We applied a self-designed, semi-structural questionnaire to collect basic demographic information [e.g., age, body mass index (BMI), number of children, cigarette/alcohol consumption] and cultural characteristics (e.g., ethnicity, religion, marital status, daily labor time, education, occupation, personal income, whether they were in charge of financial matters, family decision-making style, and family status). Family decision-making style was measured by asking participants how the family made decisions, especially when family members had differing opinions. If there was a clear decision maker, the family decision-making style would be considered as male-dominated or female-dominated according to the gender of the decision-maker. If there was no single decision maker, the family decision-making style would be considered as co-deliberative. Family status was evaluated by the Cantril ladder (Figure 1; Goodman et al., 2001; Lu et al., 2014), in which 01 represents the lowest status in the family, and 10 represents the highest status in the family. Participants were asked to report their status in the private family sphere according to their subjective perceptions. Among these items, education, occupation, personal income, whether they were in charge of financial matters, and family decision-making style could comprehensively reflect the objective dimension of women’s status. Particularly, the family decision-making style, which represents the family power, is one of the main objective dimensions of family status (Song and Zhang, 2021).

The Chinese version of the Multidimensional Scale of Perceived Social Support (MSPSS) was used to measure social support (Zimet et al., 1990). This 7-point Likert scale includes 12 items in total, ranging from 1 (strongly disagree) to 7 (strongly agree). It evaluates subjective feelings of social support from three sources, including family, friends, and other support. Thus, a higher score indicates higher social support. In this study, the Cronbach’s alpha coefficients of the family support (Items 3, 4, 8, 11), friend support (Items 6, 7, 9, 12), and other support (Items 1, 2, 5, 10) subscales were 0.832, 0.868, and 0.796, respectively.

To measure the severity of menopause-related symptoms, we used the 11-item Menopause Rating Scale (MRS) (Heinemann et al., 2003). It contains three subscales evaluating urogenital, somatic, and psychological symptoms on 5-point Likert scales, ranging from 0 (none) to 4 (very severe). A higher score indicates more severe menopause symptoms. The scale has high internal consistency, with Cronbach’s alphas ranging from 0.83 to 0.87 (Heinemann et al., 2004). In this study, the internal consistency reliability was high, with a Cronbach’s alpha of 0.84.

### Data Analysis

This study used SPSS 20.0 for statistical analyses. ANOVA and χ^2^-tests were used to compare sociodemographic characteristics, and scores of MSPSS and MRS among Mosuo, Yi, and Han women. Multiple linear regression was used to determine potential factors associated with menopause symptoms, and *p* < 0.05 two-sided was considered to be statistically significant. For all analyses, partial η^2^ and Cramer’s *V* were used as measures of effect size for ANOVA and χ^2^-tests, respectively. Cohen’s criteria to classify the effect sizes was used; small effect: partial η^2^ = 0.01; medium effect: partial η^2^ = 0.06; and large effect: partial η^2^ = 0.14 (Cohen, 1988; Kroesbergen et al., 2014).

## Results

### Sociodemographic Characteristics

Table 1 shows the demographic characteristics of the Mosuo, Yi, and Han women. Most of the participants were farmers. Compared with Mosuo and Han women, Yi women had significantly lower scores of BMI (*P* = 0.025), lower education years and level (*P* < 0.001), and more children (*P* = 0.020). The Yi women also had higher rates of smoking and drinking than their Han counterparts. As for religion, most Mosuo women (79.8%) followed Vajrayana Buddhism and Daba, most Yi women (86.2%) had faith in SuNi or BiMo (their folk gods), and only 6.8% Han women pursued any religion (*P* < 0.001). In terms of the family situation, considerable differences were noted in marital status, family status, and decision-making style among these three groups. A great amount of Mosuo (59.5%) preferred “walking marriage” (*P* < 0.001), and had significantly higher level of family status than Yi and Han women (*P* = 0.034). Mosuo women had significantly higher rates of female-dominated family decision-making style (26.2%) than Yi (13.8%) and Han women (8.6%) (*P* = 0.010).

**TABLE 1:** Sociodemographic characteristics.

Variables	Mosuo (*n* = 84) Mean ± *SD* or *N* (%)	Yi (*n* = 65) Mean ± *SD* or *N* (%)	Han (*n* = 59) Mean ± *SD* or *N* (%)	*F/X^2^*	Partial.η*^2^/*Cramer’s *V*
Age (yr)	50.20 ± 5.06	49.32 ± 5.53	49.34 ± 4.63	0.73	0.007
BMI	23.00 ± 3.61	21.54 ± 3.34	22.86 ± 3.13	3.75*	0.035
Religion				102.68***	0.703
None	17 (20.2)	9 (13.8)	55 (93.2)		
Education				10.99***	0.230
Uneducated	62 (73.8)	61 (93.8)	44 (74.6)		
Education, years	1.63 ± 3.36	0.23 ± 1.08	1.76 ± 3.19	5.98**	0.055
Occupation				4.44	0.146
Farmers	79 (94.05)	58 (89.23)	49 (83.05)		
Income (RMB^a^/yr/person)	6296.61 ± 5285.89	4661.32 ± 4269.69	6709.3 ± 6828.89	1.99	0.025
Control money				0.62	0.054
Yes	62 (73.8)	46 (70.8)	40 (67.8)		
Labor time (h/day)	7.83 ± 2.76	8.89 ± 3.15	7.97 ± 2.79	2.60	0.025
Marital status				106.00***	0.491
Traditional Mosuo marriage^b^	31 (36.9)				
Modern Mosuo marriage^c^	19 (22.6)				
Traditional marriage^d^	30 (35.7)	60 (92.3)	56 (94.9)		
Divorced	1 (1.2)		1 (1.7)		
Widowed	3 (3.6)	5 (7.7)	2 (3.4)		
Number of children	2 ± 1	3 ± 1	2 ± 1	4.00*	0.007
Family status of female	8.13 ± 2.16	7.29 ± 1.94	7.88 ± 1.66	3.43*	0.032
Family decision-making style				13.30*	0.179
Female-dominated	22 (26.2)	9 (13.8)	5 (8.6)		
Male-dominated	5 (6.0)	13 (20.0)	8 (13.8)		
Co-deliberative	57 (67.9)	43 (66.1)	45 (77.6)		
Smoking or drinking				7.02*	0.184
Yes	29 (34.5)	26 (40.0)	11 (18.6)		
No	55 (65.5)	39 (60.0)	48 (81.4)		

*^a^RMB, Ren Min Bi; currency in China.*

*^b^Traditional Mosuo marriage, also known as “walking marriage.”*

*^c^Modern Mosuo marriage, also known as “walking marriage.”*

*^d^Traditional marriage, Monogamous marital relationship. One man and one woman were married and living together in a nuclear family.*

*P < 0.05; **P < 0.01; ***P < 0.001.

*BMI, body mass index; yr, year; SD, Standard Deviation; h, hour.*

### Comparison of Multidimensional Scale of Perceived Social Support Among the Mosuo, Yi, and Han Women Groups

Table 2 presents the differences of the MSPSS subscale among the Mosuo, Yi, and Han women groups. There were no significant differences of friend and other support subscales among Mosuo, Yi, and Han women. There were significant differences of family support scores among three women groups [*F*(2, 203) = 3.81, *P* = 0.024]. *Post-hoc* tests showed that the Mosuo women group had significantly higher scores on family support than the Yi women group (*MD* = 2.10, *P* = 0.007).

**TABLE 2:** Comparison of MSPSS subscale scores among Mosuo, Yi, and Han women.

Items	Mosuo (*n* = 84) Mean ± *SD*	Yi (*n* = 65) Mean ± *SD*	Han (*n* = 59) Mean ± *SD*	*F*	Partial.η*^2^*
MSPSSfa	24.89 ± 3.69	22.80 ± 5.43	23.68 ± 4.82	3.81**	0.036
MSPSSfr	22.80 ± 5.24	21.22 ± 6.96	23.36 ± 3.92	2.55	0.024
MSPSSoth	19.71 ± 6.67	19.27 ± 6.10	17.56 ± 6.72	1.99	0.019

*MSPSS, Multidimensional Scale of Perceived Social Support; MSPSSfa, perceived social support from family; MSPSSfr, perceived social support from friends; MSPSSoth, perceived social support from others.*

**P < 0.01.

### Comparison of the Menopause-Related Symptoms Among Mosuo, Yi, and Han Women Groups

To eliminate confounding bias caused by villages, diagnostic tests through ANOVA were conducted. Results showed no difference among the villages [*F*(2, 202) = 1.66, *P* = 0.139], indicating that MRS differences were not caused by the participants coming from different villages. Table 3 shows the comparison of the menopause-related symptoms measured by the MRS among Mosuo, Yi, and Han women groups. The results of ANOVA with Bonferroni correction showed that the total MRS scores were significantly different among Mosuo, Yi, and Han women groups [*F*(2, 202) = 6.09, *P* = 0.003]. *Post hoc* tests showed that the total MRS scores in Mosuo women were significantly lower than those in Yi (*MD* = –3.89, *P* = 0.006) and Han women (*MD* = –3.46, *P* = 0.021). The total MRS scores were also significantly different among the three ethnic women groups in psychological [*F*(2, 202) = 4.38, *P* = 0.014)], somatic [*F*(2, 202) = 5.79, *P* = 0.004)], and urogenital symptoms [*F*(2, 202) = 3.13, *P* = 0.046)]. The psychological scores of the MRS in Mosuo women were significantly lower than those in Han women (*MD* = –1.59, *P* = 0.018), and the somatic scores of the MRS in Mosuo women were significantly lower than those in Yi women (*MD* = –1.87, *P* = 0.003).

**TABLE 3:** Comparison of the MRS scores among Mosuo, Yi, and Han women.

Items	Mosuo (*n* = 84) Mean ± *SD*	Yi (*n* = 65) Mean ± *SD*	Han (*n* = 59) Mean ± *SD*	*F*	Partial.η^2^
Hot flashes, sweating (episodes of sweating)	0.89 ± 1.15	1.13 ± 1.11	1.02 ± 1.18	0.77	0.008
Heart discomfort (unusual awareness of heartbeat, heart skipping, heart racing, tightness)	0.87 ± 1.07	1.5 ± 1.25	0.97 ± 1.05	6.11**	0.057
Sleep problems (difficulty falling asleep, difficulty sleeping through the night, waking up early)	0.54 ± 0.86	1.16 ± 1.31	1.14 ± 1.12	7.91***	0.073
Depressive mood (feeling down, sad, on the verge of tears, lack of drive, mood swings)	0.70 ± 0.93	0.98 ± 1.08	1.05 ± 1.07	2.43	0.023
Irritability (feeling nervous, inner tension, feeling aggressive)	0.82 ± 1.08	1.01 ± 1.01	1.15 ± 1.08	1.76	0.017
Anxiety (inner restlessness, feeling panicky)	0.79 ± 1.03	1.10 ± 1.25	1.08 ± 1.12	1.82	0.018
Physical and mental exhaustion (general decrease in performance, impaired memory, decrease in concentration, forgetfulness)	0.80 ± 1.18	1.11 ± 1.32	1.32 ± 1.11	3.32*	0.032
Sexual quality (decreased libido, decreased sexual activity, dissatisfaction)	0.72 ± 1.24	0.92 ± 1.24	1.12 ± 1.27	1.79	0.018
Bladder problems (difficulty urinating, increased need to urinate, bladder incontinence)	0.29 ± 0.74	0.48 ± 0.88	0.47 ± 0.88	1.37	0.013
Dryness of vagina (sensation of dryness or burning in the vagina, difficulty with sexual intercourse)	0.32 ± 0.99	0.69 ± 1.18	0.56 ± 1.12	2.11	0.021
Joint and muscular discomfort (pain in the joints, rheumatoid complaints)	1.33 ± 1.10	1.84 ± 1.28	1.58 ± 1.13	3.35*	0.032
Total score	8.00 ± 6.84	11.89 ± 7.69	11.46 ± 8.03	6.09**	0.057
Psychologic score	3.02 ± 3.10	4.21 ± 3.64	4.61 ± 3.45	4.38*	0.042
Somatic score	3.64 ± 3.09	5.52 ± 3.59	4.69 ± 3.36	5.79**	0.054
Urogenital score	1.26 ± 2.15	1.95 ± 2.26	2.15 ± 2.41	3.13*	0.030

*P < 0.05; **P < 0.01; ***P < 0.001.

*MRS, Menopause rating scale.*

Some of these symptoms showed significant differences between groups with small to moderate effect size *(partial*η^2^), which suggested the commonality of some symptoms among Mosuo, Yi and Han women. Among the three ethnic groups, muscle and joint pain were reported as the most severe, and bladder problem was the least severe. Yi women complained of more heart symptoms than Mosuo (*MD* = 0.63, *P* = 0.003) and Han women (*MD* = 0.53, *P* = 0.029). Mosuo women had significantly lower sleep scores than Yi (*MD* = 0.63, *P* = 0.002) and Han women (*MD* = 0.60, *P* = 0.004). Yi women had significantly higher scores in joint and muscle discomfort than Mosuo women (*MD* = 0.51, *P* = 0.031). Regarding physical and mental exhaustion, Han women reported significantly more fatigue than Mosuo women (*MD* = 0.51, *P* = 0.037).

### Influencing Factors Associated With the Menopause-Related Symptoms

Table 4 shows the influencing factors associated with the menopause-related symptoms using the multiple linear regression analysis. Based on the results of bivariate analysis, variables including ethnicity, age, daily labor time, family decision-making style, family status, and family support, were entered in the regression analysis. The results showed that the average daily labor hours were not significantly associated with the menopause-related symptoms in middle-aged women (β = 0.273, *P* = 0.091). Ethnicity, age, family support, and family decision-making patterns were significantly associated with the menopause-related symptoms in middle-aged women (*R*^2^ = 0.322). Compared with Mosuo, it was found that Yi and Han women had significantly more menopause-related symptoms. Age had a significant negative impact on the severity of menopause-related symptoms. The family support experienced by participants had a significant positive impact on the severity of menopause-related symptoms. The women living in the “female-dominated” and the “co-deliberative” family had significantly less menopause-related symptoms than those living in the “male-dominated” family. There were no significant associations between family support and ethnicity (*F* = 1.26, *P* = 0.285), or family status and ethnicity (*F* = 1.54, *P* = 0.172).

**TABLE 4:** Influencing factors associated with the MRS.

Model			Unstandardized coefficients		Standardized coefficients			Sig.	Collinearity statistics
						
			B	Std. error	Beta				VIF
Independent variables	(Constant)		5.433	5.738		0.947		0.345	
	Ethnicity	Yi	3.090	1.165	0.182	2.652		0.009	1.322
		Han	3.330	1.138	0.198	2.927		0.004	1.285
		Mosuo	0.00						
	Age		0.265	0.093	0.175	2.865		0.005	1.048
	MSPSSfa*^b^*		–0.260	0.103	–0.160	–2.536		0.012	1.116
	Family decision-making style	Co-deliberative	–8.107	1.430	–0.489	–5.669		0.000	2.088
		Female-dominated	–4.295	1.725	–0.213	–2.490		0.014	2.055
		Male-dominated	0						
*R* ^2^			0.322	
Adjusted *R*^2^			0.297	
*F*			12.882	
*P*			<0.001	

*Dependent Variable: MRS; MSPSSfa, perceived social support from family.*

## Discussion

This is the first study to investigate the menopause-related symptoms in middle-aged women in Mosuo, Yi, and Han ethnic groups. This study indicated that Mosuo women had significantly higher levels of family status and family support than Yi and Han women. The results showed that the three ethnic groups had different levels of menopause-related symptoms. Generally, Mosuo women reported significantly less menopause-related symptoms than Yi and Han women. Ethnicity, age, family support, and family decision-making style were significantly associated with the menopause-related symptoms. Moreover, family support and family decision-making style were also significantly associated with the menopause-related symptoms in middle-aged women, regardless of their ethnicities. The results are consistent with previous epidemiological studies that Mosuo women generally have better mental health than other ethnic groups (Xu et al., 2018; Yang et al., 2018). For instance, the incidence of depression during perimenopause in Mosuo women is lower than that in Han women (Zhang et al., 2013, 2019).

The results of this study indicate that different ethnic women groups have various levels of menopause-related symptoms. Due to the regional segregation, ethnic groups in China living in separate villages but near each other, had varied traditional customs and cultures, although they shared similar income level, economic activities, and geographical environment (Gong and Yang, 2012). The possible reasons why Mosuo women had significantly lower levels of menopause-related symptoms than Yi and Han women may be related to the following: First, the women’s family status may be partly related with cultural background, such as their matrilineal culture. This study indicates that Mosuo women have higher family status (e.g., more decision-making power) and are respected in the family and community (Zhang et al., 2013, 2020; He et al., 2015; Yang, 2020). So, Mosuo women may have less pressure to have more children, and do not report menopause-related symptoms. A study in India also reported that Indian women of the Rajput caste did not report any symptoms associated with menopause, because they were rewarded for reaching the menopausal state. They would be given a higher status than when they were reproductive (Flint and Samil, 1990). Similarly, Mosuo women enjoyed a high family status to begin with and were exceedingly respected as the core of the family, and even of the society (Zhang et al., 2013, 2020; He et al., 2015; Yang, 2020). This study once again confirmed that Mosuo women’s family status and family support were significantly higher than that of Yi women. Mosuo women have more decision-making power during a situation of conflict among family members. They inherit a higher social status as they enjoy the traditional values of “respecting females and worshiping mother.” However, Mosuo women do not show significant differences from other ethnic groups in occupational status, working hours, and income. The results of multiple linear regression also suggest that family decision-making style and family support were predictors of the lower severity of menopause-related symptoms, consistent with the previous study that reveals that lower socioeconomic status (SES) was associated with longer duration and increased severity of the menopausal symptoms (Blümel et al., 2006). Second, evidence shows that attitudes toward menopause are related to menopausal symptoms (Yanikkerem et al., 2012). In this study, Mosuo women often use “clean, convenient, and peaceful” to describe the cessation of their menstruation. They often have positive attitudes toward life changes, such as “let it be” and “do not think too much,” to help them relax and maintain family unity and harmony (Yang, 2013; Yan and Song, 2016). This may have a protective effect on Mosuo women’s physical and mental health (e.g., to have less menopause-related symptoms). Further studies should be conducted to examine this.

This study showed that the most prominent symptoms of Yi women were somatic complaints, especially pain in the joints with visible joint deformation. Compared with Mosuo and Han women, Yi women had a lower BMI, more children, and lower education level. As most Yi men work far away from their homes, the women have less opportunity to get support from their family members (e.g., husband) at their homes. When Yi women suffer from illness, they are more likely to resort to alcohol, painkillers, and seek help from village doctors rather than seeing a doctor in the hospital. The possible reasons may be related to Yi women’s lower socioeconomic status (e.g., many Yi people live in mountain areas), lack of knowledge regarding health, and limited access to health services or information (Lynch et al., 2000; Phelan et al., 2010; Lim et al., 2019).

The possible reasons for higher level of menopause-related symptoms in Han women may be associated with the following: Evidence shows that the severity of menopause-related psychological symptoms in Han people may be associated with the conceptualization of menopause being characterized by irritable outbursts (Shea, 2020). The results of this study indicated that Han women were less likely to have religious beliefs than Yi and Mosuo women. Previous studies suggested that religious involvement might be a powerful protective resource for emotional problems (Koenig et al., 2020). Therefore, the religious beliefs of the Mosuo and Yi women may play an important protective role in their midlife, which is absent in the Han women. Moreover, Han women may be more stressed than Mosuo women during the period of middle-age, as they still have to endeavor to maintain their family socioeconomic status.

In general, middle-aged women are at higher risks of various menopause-related symptoms and psychological disturbances (Deeks, 2004; Li and Graham, 2017). These symptoms are related to biological, psychological, social, and cultural factors (Namazi et al., 2019). In this study, higher level of family support and lower level of menopausal symptoms among the Mosuo women suggest that family support has a culture-specific protective effect on middle-aged women’s physical and mental health (e.g., menopause-related symptoms). Furthermore, this study also suggests that being respected and supported are beneficial for the individual’s physical and mental health (Fasihi Harandi et al., 2017).

### Contributions and Implications

Our study reveals the interrelation between health and cultural context. First, the menopause-related symptoms of middle-aged women are strongly influenced by their ethnic background, family status, and family support. Second, from the perspective of psychosomatic medicine and cultural psychiatry, this study explored the individual’s psychological experience, life situation, and cultural factors related to menopause-related symptoms. Third, this study provides evidence on the impact of the cultural context on an individual’s health problems (e.g., menopause-related symptoms) in different ethnic women groups. This will be helpful for planning psychosocial interventions, and providing effective health care services for middle-aged women in different ethnic groups.

### Limitations and Future Directions

This study also has some limitations. First, this is a cross-sectional investigation, and the causal relationship cannot be explored. Second, the sample size is relatively small; larger-sample size studies should be conducted in the future to confirm the current results. Third, snowball sampling may limit the generalizability. In order to minimize the sampling biases, we used the same sampling method across all three ethnic groups. Fourth, even though we recruited local interpreters, due to language barriers among the participants, in-depth interviews with them were limited. The significance of the menopause-related symptoms in different cultures should be explored further. More studies including qualitative and quantitative approaches should be conducted, to obtain more robust and compelling evidence by interviewing the subjects and their families.

## Data Availability Statement

The original contributions presented in the study are included in the article, further inquiries can be directed to the corresponding author/s.

## Ethics Statement

The studies involving human participants were reviewed and approved by Ethics Committee of Shanghai Pudong New Area Mental Health Center. Written informed consent for participation was not required for this study in accordance with the national legislation and the institutional requirements.

## Author Contributions

JW, LG, and XZ conceived of the presented idea. JW developed the theory and designed the research protocol. JW, XL, and CH collected the data. JW and XL verified the analytical methods. XZ supervised the findings of this work. MR participated in manuscript revision and editing and was involved in the manuscript development. All authors discussed the results and contributed to the final manuscript.

## Conflict of Interest

The authors declare that the research was conducted in the absence of any commercial or financial relationships that could be construed as a potential conflict of interest.

## Publisher’s Note

All claims expressed in this article are solely those of the authors and do not necessarily represent those of their affiliated organizations, or those of the publisher, the editors and the reviewers. Any product that may be evaluated in this article, or claim that may be made by its manufacturer, is not guaranteed or endorsed by the publisher.

## References

[B1] Abasi E. Keramat A. Ghorbani M. (2020). The Relationship between Social Support, General Health Status, and Severity of Menopause Symptoms among Postmenopausal Women in Northern Iran. *J. Med. Heal. Sci.* 14 771–776.

[B2] Avis N. E. Stellato R. Crawford S. Bromberger J. Ganz P. Cain V. (2001). Is there a menopausal syndrome? Menopausal status and symptoms across racial/ethnic groups. *Soc. Sci. Med.* 52 345–356. 10.1016/s0277-9536(00)00147-7 11330770

[B3] Begum K. Muttukrishna S. Sievert L. L. Sharmeen T. Murphy L. Chowdhury O. (2016). Ethnicity or environment: effects of migration on ovarian reserve among Bangladeshi women in the United Kingdom. *Fertil. Steril.* 105 744–754. 10.1016/j.fertnstert.2015.11.024 26706133

[B4] Blümel J. E. Chedraui P. Calle A. Bocanera R. Depiano E. Figueroa-Casas P. (2006). Age at menopause in Latin America. *Menopause* 13 706–712. 10.1097/01.gme.0000227338.73738.2d 16837893

[B5] Cai H. (2001). *A Society Without Fathers or Husbands the Na of China.* New York, NY: Zone Books

[B6] Chinese Medical Doctor Association (2021). Expert Consensus on Menopausal Women’s Health Management (Basic Edition) (in Chinese). *J. Appl. Clin. Pediat.* 24 1317–1324.

[B7] Cohen J. (1988). *The Effect size. Statistical Power Analysis for the Behavioral Sciences*, 2nd Edn. Hillsdale, NJ: Lawrence Erlbaum Associates, 77–83.

[B8] Deeks A. A. (2004). Is this menopause?: women in midlife-psychosocial issues. *Aust. Fam. Phys.* 33 889–893. 15584326

[B9] El Khoudary S. R. Greendale G. Crawford S. L. Avis N. E. Brooks M. M. Thurston R. C. (2019). The menopause transition and women’s health at midlife: a progress report from the Study of Women’s Health Across the Nation (SWAN). *Menopause* 26 1213–1227. 10.1097/GME.0000000000001424 31568098 PMC6784846

[B10] Facchinetti F. Demyttenaere K. Fioroni L. Neri I. Genazzani A. R. (1992). Psychosomatic disorders related to gynecology. *Psychother. Psychosomat.* 58 137–154. 10.1159/000288622 1488498

[B11] Fasihi Harandi T. Mohammad Taghinasab M. Dehghan Nayeri T. (2017). The correlation of social support with mental health: a meta-analysis. *Electron. Phys.* 9 5212–5222. 10.19082/5212 29038699 PMC5633215

[B12] Faul F. Erdfelder E. Lang A. G. Buchner A. (2007). G* Power 3: a flexible statistical power analysis program for the social, behavioral, and biomedical sciences. *Behav. Res. Methods* 39 175–191. 10.3758/bf03193146 17695343

[B13] Feng L. (2013). *Early China: A Social and Cultural History.* Cambridge: Cambridge University Press.

[B14] Flint M. (1975). The menopause: reward or punishment? *Psychosomatics* 16 161–163. 10.1016/S0033-3182(75)71183-0 1197605

[B15] Flint M. Samil R. S. (1990). Cultural and subcultural meanings of the menopause. *Annals N Y. Acad. Sci.* 592 134–148. 10.1111/j.1749-6632.1990.tb30321.x 2197940

[B16] Gong B. Yang C. (2012). Gender differences in risk attitudes: field experiments on the matrilineal Mosuo and the patriarchal Yi. *J. Econ. Behav. Organ.* 83 59–65. 10.1016/j.jebo.2011.06.010

[B17] Goodman E. Adler N. E. Kawachi I. Frazier A. L. Huang B. Colditz G. A. (2001). Adolescents’ perceptions of social status: development and evaluation of a new indicator. *Pediatrics* 108:E31. 10.1542/peds.108.2.e31 11483841

[B18] Greendale G. A. Lee N. P. Arriola E. R. (1999). The menopause. *Lancet* 353 571–580. 10028999 10.1016/S0140-6736(98)05352-5

[B19] Guo J. (2017). *The Study on the Changes and Development of Mosuo Culture.* Kunming: Yunnan University.

[B20] Halbreich U. Karkun S. (2006). Cross-cultural and social diversity of prevalence of postpartum depression and depressive symptoms. *J. Affect. Disord.* 91 97–111. 10.1016/j.jad.2005.12.051 16466664

[B21] Hao Y. (2015). The status of women in yi family culture (in Chinese). *J. Southwest Univ. Nat.* 36 55–59.

[B22] Harrell S. (2001). *Ways of Being Ethnic in Southwest China.* Seattle, WA: University of Washington Press.

[B23] HasErdene (2018). From “Tatar” to “Mosuo”: the winding path of an ethnic minority identity in China. *Asian Anthropol.* 17 221–232. 10.1080/1683478X.2018.1501836

[B24] He X. Zhang X. Zhang J. Xiao E. Wang J. (2015). Cultural Schema Affect the Spatial Metaphors in the Semantic Processing of Kinship Words: The Evidence from the Han and the Moso (in Chinese). *Acta Psychol. Sin.* 47 584–599. 10.3724/sp.j.1041.2015.00584

[B25] Heinemann K. Ruebig A. Potthoff P. Schneider H. P. Strelow F. Heinemann L. A. (2004). The Menopause Rating Scale (MRS) scale: a methodological review. *Health Q. Life Outcome* 2:45. 10.1186/1477-7525-2-45 15345062 PMC516787

[B26] Heinemann L. A. Potthoff P. Schneider H. P. (2003). International versions of the menopause rating scale (MRS). *Health Q. Life Outcomes* 1:28. 10.1186/1477-7525-1-28 12914663 PMC183844

[B27] Hvas L. Thorsen H. Søndergaard K. (2003). Discussing menopause in general practice. *Maturitas* 46 139–146. 10.1016/s0378-5122(03)00164-6 14559385

[B28] Koenig H. G. Al-Zaben F. VanderWeele T. J. (2020). Religion and psychiatry: recent developments in research. *BJPsych. Adv.* 26 262–272. 10.1192/bja.2019.81

[B29] Kroesbergen E. H. van’t Noordende J. E. Kolkman M. E. (2014). Training working memory in kindergarten children: effects on working memory and early numeracy. *Child Neuropsychol.* 20 23–37. 10.1080/09297049.2012.736483 23098260

[B30] Li S. H. Graham B. M. (2017). Why are women so vulnerable to anxiety, trauma-related and stress-related disorders? The potential role of sex hormones. *Lancet Psychiatry* 4 73–82. 10.1016/S2215-0366(16)30358-3 27856395

[B31] Lim Y. Jeong K. Lee S. R. Chung H. W. Lee W. (2019). Association between premature ovarian insufficiency, early menopause, socioeconomic status in a nationally representative sample from Korea. *Maturitas* 121 22–27. 10.1016/j.maturitas.2018.12.004 30704561

[B32] Liu Z. (2007). *Educational Anthropology Research on the Inheritance of Yi Nationality Family Culture in Liangshan.* Beijing: Minzu University of China.

[B33] Lu X. Guo Y. Li J. (2014). Social Class and Prosocial Behavior: The Moderating Effects of Return Prediction. *J. Psychol. Sci.* 37 1212–1219. 10.16719/j.cnki.1671-6981.2014.05.036

[B34] Lynch J. W. Smith G. D. Kaplan G. A. House J. S. (2000). Income inequality and mortality: importance to health of individual income, psychosocial environment, or material conditions. *BMJ* 320 1200–1204. 10.1136/bmj.320.7243.1200 10784551 PMC1127589

[B35] Melby M. K. Lock M. Kaufert P. (2005). Culture and symptom reporting at menopause. *Hum. Reproduct. Update* 11 495–512. 10.1093/humupd/dmi018 15919681

[B36] Movahed E. Pourreza A. Rahimi Foroshani A. (2015). The effect of social support on the health of the elderly in Tehran. *J. Hospital* 13 115–121.

[B37] Namazi M. Sadeghi R. Moghadam Z. B. (2019). Social determinants of health in menopause: an integrative review. *Int. J. Women’s Health* 11 637–647. 10.2147/IJWH.S228594 31849539 PMC6910086

[B38] Obermeyer C. M. (2000). Menopause Across Cultures: A Review of the Evidence. *Menopause* 7 184–192. 10.1097/00042192-200007030-00009 10810964

[B39] Parkerson G. R. Jr. Michener J. L. Wu L. R. Finch J. N. Muhlbaier L. H. Magruder-Habib K. (1989). Associations among family support, family stress, and personal functional health status. *J. Clin. Epidemiol.* 42 217–229. 10.1016/0895-4356(89)90058-9 2785165

[B40] Phelan J. C. Link B. G. Tehranifar P. (2010). Social conditions as fundamental causes of health inequalities: theory, evidence, and policy implications. *J. Health Soc. Behav.* 51 S28–S40. 10.1177/0022146510383498 20943581

[B41] Shea J. L. (2006). Cross-cultural comparison of women’s midlife symptom-reporting: A China study. *Cult. Med. Psychiatry* 30 331–362. 10.1007/s11013-006-9020-4 17048096

[B42] Shea J. L. (2020). Menopause and Midlife Aging in Cross-Cultural Perspective: Findings from Ethnographic Research in China. *J. Cross Cult. Gerontol.* 35 367–388. 10.1007/s10823-020-09408-6 32779059

[B43] Song J. Zhang X. (2021). Measurement of Women’s Status inside the Family. *J. Shandong Women’s University* 1, 1–10.

[B44] Sum C.-Y. Blumenfield T. Shenk M. K. Mattison S. M. (2021). Hierarchy, Resentment, and Pride: Politics of Identity and Belonging among Mosuo, Yi, and Han in Southwest China. *Modern China* 48 568–592. 10.1177/00977004211017814

[B45] WHO Scientific Group (1996). Research on the menopause in the 1990s. *World Health Organ. Tech. Rep. Series* 866 1–107. 8942292

[B46] Wu F. Sheng Y. (2019). Social support network, social support, self-efficacy, health-promoting behavior and healthy aging among older adults: a pathway analysis. *Arch. Gerontol. Geriatrics* 85:103934. 10.1016/j.archger.2019.103934 31466024

[B47] Xu L. Wang Q. Yang J. Lin Y. Zhang H. Zeng X. (2018). Epidemiological survey of major depressive disorder and dysthymia among Mosuo people in Ninglang district, Yunnan Province (in Chinese). *Chin. J. Behav. Med. Brain Sci.* 27 758–762.

[B48] Yan M. Song P. (2016). On the Mosuo Traditional View of Death and Hospice Care (in Chinese). *J. Xizang University* 31 16–21. 10.16249/j.cnki.1005-5738.2016.03.003

[B49] Yan R. (1984). A living fossil of the family: a study of the family structure of the Naxi nationality in the Lugu Lake region. *Soc. Sci. Chin.* 3 60–83.

[B50] Yang J. Zeng X. Yang J. Zhong H. Lin Y. Zhang H. (2018). Epidemiological investigation of obsessive-compulsive disorder in Mosuo people of Ninglang area (in Chinese). *J. Clin. Psychol. Med.* 28 57–59.

[B51] Yang L. (2013). Ethnic Tourism and Minority Identity: Lugu Lake, Yunnan, China. *Asia Pacific J. Tour. Res.* 18 712–730. 10.1080/10941665.2012.695289

[B52] Yang S. (2020). *Legal Thinking on the Marriageand Family Relationship of Mosuopeople——A Case Study of Mosuo,Yongning Township, NinglangCounty, Yunnan Province.* Kunming: Yunnan Normal University.

[B53] Yanikkerem E. Koltan S. O. Tamay A. G. Dikayak Ş (2012). Relationship between women’s attitude towards menopause and quality of life. *Climacteric* 15 552–562. 10.3109/13697137.2011.637651 22335298

[B54] Zhang H. Yan J. (2021). *Environment and Female Reproductive Health.* Berlin: Springer.

[B55] Zhang J. Fu Y. Wang B. (2020). Gender culture influence on spatial and weight metaphors of kinship words: Evidence from Bai, Yi, and Mosuo nationalities (in Chinese). *Acta Psychol. Sin.* 52 440–455. 10.3724/SP.J.1041.2020.00440

[B56] Zhang Y. Wang J. Zhao Y. Zhao X. (2013). Somatic and psychological symptoms and help-seeking behaviors between Mosuo and Han Chinese women during climacteric stage (in Chinese). *Chin. Mental Health J.* 27 686–691.

[B57] Zhang Y. Zhao X. Leonhart R. Nadig M. Wang J. Zhao Y. (2019). A Cross-Cultural Comparison of Climacteric Symptoms, Health-Seeking Behavior, and Attitudes towards Menopause Among Mosuo Women and Han Chinese Women in Yunnan, China. *Transcult. Psychiatry* 56 287–301. 10.1177/1363461518804094 30444458

[B58] Zhao D. Liu C. Feng X. Hou F. Xu X. Li P. (2019). Menopausal symptoms in different substages of perimenopause and their relationships with social support and resilience. *Menopause* 26 233–239. 10.1097/GME.0000000000001208 30252803

[B59] Zhao P. (2011). *The Contemporary Situation of Walking Marriage around Lugu Lake.* Beijing: Minzu University of China.

[B60] Zheng W. (2020). *Women in the Chinese enlightenment.* Berkeley: University of California Press.

[B61] Zhou X. Peng Y. Zhu X. Yao S. Dere J. Chentsova-Dutton Y. E. (2016). From culture to symptom: testing a structural model of “Chinese somatization”. *Transcult. Psychiatry* 53 3–23. 10.1177/1363461515589708 26076689

[B62] Zimet G. D. Powell S. S. Farley G. K. Werkman S. Berkoff K. A. (1990). Psychometric characteristics of the Multidimensional Scale of Perceived Social Support. *J. Personal. Assess.* 55 610–617. 10.1080/00223891.1990.9674095 2280326

